# Supplementary material for: Risk, Diagnostic and Predictor Factors for Classical Hodgkin Lymphoma in HIV-1-Infected Individuals: Role of Plasma Exosome-Derived miR-20a and miR-21
Source: J Clin Med. 2020 Mar 11;9(3):760. doi: 10.3390/jcm9030760 (PMC7141191; doi:10.3390/jcm9030760)
Supplement: Supplementary file 1 [file jcm-09-00760-s001.pdf]

Supplementary Figure S1

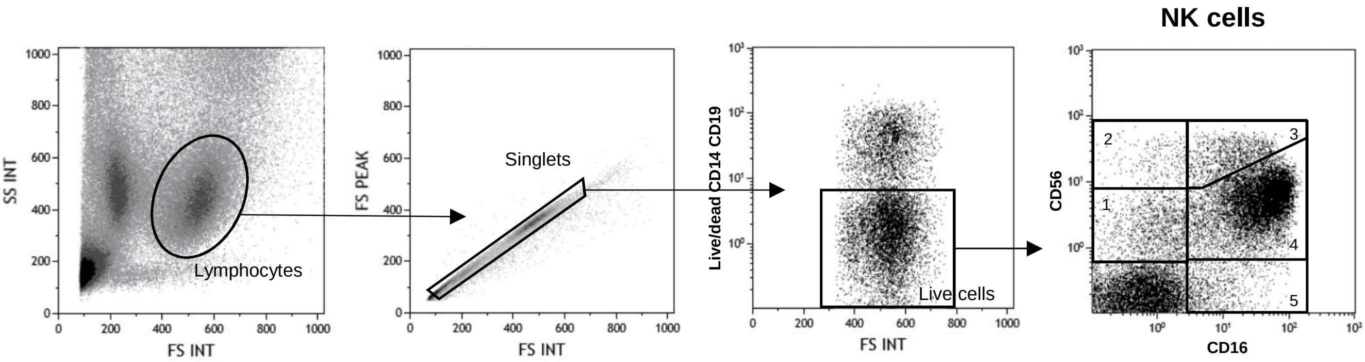

Representative sample for flow-cytometric gating strategy for NK cells. After initially gating lymphocytes according to morphological parameters only singlets were further analyzed. Only live cells CD14- and CD19- were analyzed using CD56 and CD16. NK cells were name as follows: 1, CD56dimCD16-; 2, CD56bCD16-; 3, CD56bCD16+; 4, CD56dimCD16+; and 5 CD56-CD16+.

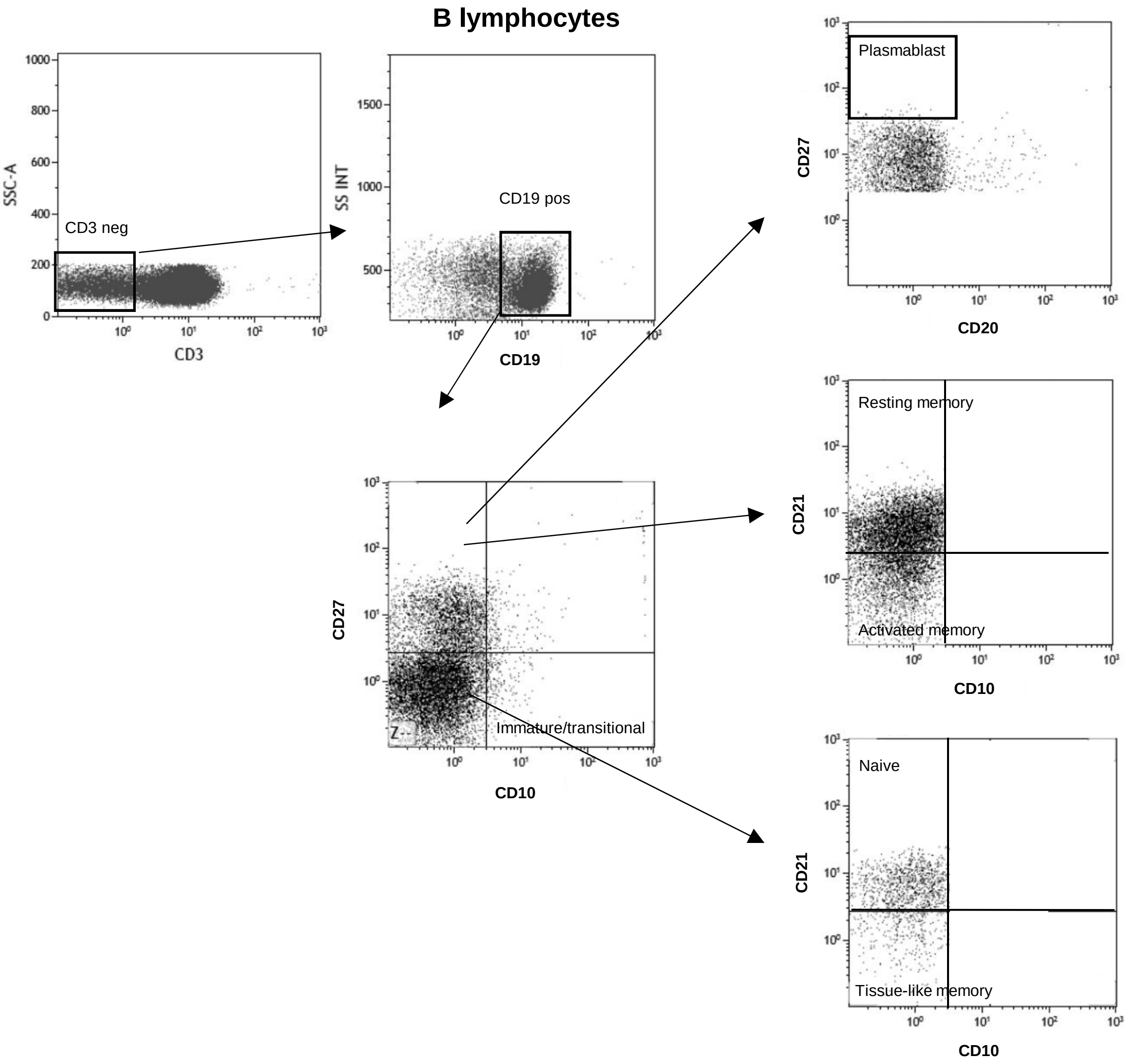

Representative sample for flow-cytometric gating strategy for B lymphocytes. After initially gating lymphocytes according to morphological parameters only singlets were further analyzed. Cells with CD3- phenotype were analyzed using CD19 (B lymphocites). B lymphocytes were name initially according to CD27 and CD10, defining Immature/transitional (CD10+CD27-) cells; Plasmablasts (CD27++CD10-CD20-), Resting memory (CD27+CD21+CD10-), Activated memory (CD27+CD21-CD10-), Naïve (CD27-CD21+CD10-), and Tissue-like memory (CD27-CD21-CD10-),

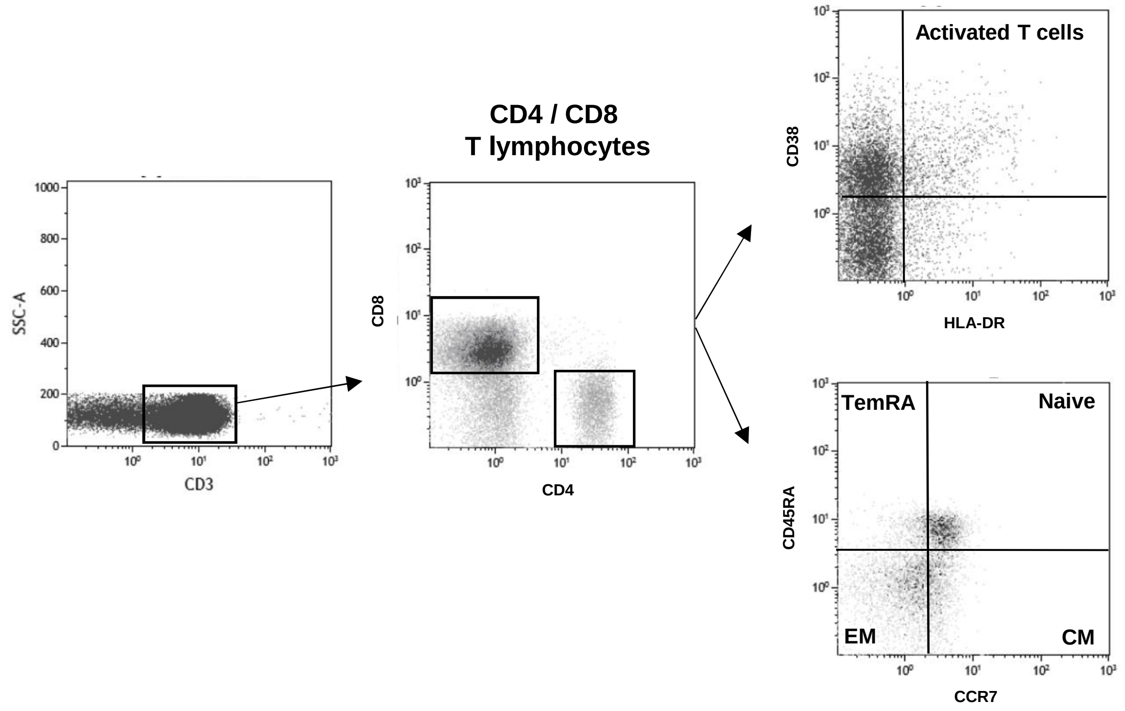

Representative sample for flow-cytometric gating strategy for T lymphocytes. After initially gating lymphocytes according to morphological parameters only singlets were further analyzed. Cells with CD3+ phenotype were analyzed using CD4 and CD8. Activated T cells were defined as the coexpression of CD38 and HLA-DR. T cell subsets were defined as: naïve (CD45RA+CCR7+), central memory (CM, CD45RA-CCR7+), effector memory (EM, CD45-CCR7-) and TemRA (CD45RA+CCR7-).

Supplementary Table S1

Biological samples		Pre cHL	cHL	Post cHL
**HL group**				
Plasma	Paired samples	11	21	12
	Single samples	4	4	7
PBMC	Paired samples	3	9	5
	Single samples	3		4
**Control group**				
Plasma			25	
PBMC			9	
Time of sample collection (months)		11 [5-28]		13 [10-22]

Supplementary Table S2

	CD4 count	CD8 count	CD4/CD8 ratio	miR-20a	miR-16	miR-106a	miR-21	miR-324	miR-185	miR-223	sCD14	sCD27	sCD30	IL2R
**Nadir CD4 count**	0.398<br><0.001	-0.030<br>0.808	0.429<br><0.001	0.032<br>0.851	0.036<br>0.836	0.048<br>0.779	0.102<br>0.552	-0.152<br>0.376	0.148<br>0.390	0.043<br>0.805	-0.126<br>0.465	-0.079<br>0.647	-0.025<br>0.884	-0.182<br>0.287
**CD4 count**		0.423<br><0.001	0.609<br><0.001	-0.180<br>0.253	0.306<br>0.049	0.322<br>0.037	-0.094<br>0.553	-0.377<br>0.014	0.061<br>0.700	-0.043<br>0.788	-0.020<br>0.899	-0.143<br>0.365	-0.248<br>0.113	-0.279<br>0.074
**CD8 count**			-0.344<br>0.003	0.059<br>0.748	0.374<br>0.035	0.330<br>0.065	-0.056<br>0.763	0.003<br>0.985	0.114<br>0.533	0.162<br>0.377	0.015<br>0.934	-0.068<br>0.712	-0.130<br>0.478	-0.148<br>0.418
**CD4/CD8 ratio**				-0.463<br>0.008	0.006<br>0.974	0.073<br>0.694	-0.221<br>0.225	-0.315<br>0.079	0.026<br>0.886	-0.137<br>0.454	-0.181<br>0.322	-0.254<br>0.160	-0.199<br>0.276	-0.202<br>0.268
**miR-20a**					-0.283<br>0.069	0.217<br>0.168	0.410<br>0.007	0.194<br>0.219	0.296<br>0.057	0.191<br>0.225	0.175<br>0.269	0.358<br>0.020	0.047<br>0.770	0.136<br>0.392
**miR-16**						0.144<br>0.364	-0.067<br>0.675	-0.213<br>0.176	0.035<br>0.828	0.166<br>0.293	-0.182<br>0.250	-0.249<br>0.111	-0.374<br>0.015	-0.291<br>0.062
**miR-106a**							0.198<br>0.208	-0.337<br>0.029	0.561<br><0.001	0.430<br>0.005	-0.057<br>0.722	-0.014<br>0.930	-0.200<br>0.204	-0.223<br>0.156
**miR-21**								0.359<br>0.020	0.133<br>0.401	0.065<br>0.680	0.423<br>0.005	0.507<br>0.001	0.270<br>0.084	0.093<br>0.559
**miR-324**									-0.040<br>0.804	-0.020<br>0.898	0.140<br>0.377	0.206<br>0.190	0.292<br>0.061	0.144<br>0.363
**miR-185**										0.490<br>0.001	-0.240<br>0.125	-0.181<br>0.251	-0.228<br>0.146	-0.318<br>0.040
**miR-223**											-0.014<br>0.931	-0.154<br>0.392	-0.327<br>0.034	-0.235<br>0.134
**sCD14**												0.325<br>0.036	0.096<br>0.545	0.205<br>0.194
**sCD27**													0.567<br><0.001	0.563<br><0.001
**sCD30**														0.669<br><0.001

Supplementary Table S3. Spanish AIDS Research Network (CoRIS), BioBank and collaborating Centers.

Executive committee

Santiago Moreno, Inma Jarrín, David Dalmau, Maria Luisa Navarro, Maria Isabel González, Federico Garcia, Eva Poveda, Jose Antonio Iribarren, Félix Gutiérrez, Rafael Rubio, Francesc Vidal, Juan Berenguer, Juan González, M Ángeles Muñoz-Fernández.

Fieldwork data management and analysis

Inmaculada Jarrin, Belén Alejos, Cristina Moreno, Carlos Iniesta, Luis Miguel Garcia Sousa, Nieves Sanz Perez, Marta Rava.

BioBanK HIV Hospital General Universitario Gregorio Marañón

M Ángeles Muñoz-Fernández, Irene Consuegra Fernández.

Hospital General Universitario de Alicante (Alicante)

Esperanza Merino, Gema García, Irene Portilla, Iván Agea, Joaquín Portilla, José Sánchez-Payá, Juan Carlos Rodríguez, Lina Gimeno, Livia Giner, Marcos Díez, Melissa Carreres, Sergio Reus, Vicente Boix, Diego Torrús.

Hospital Universitario Central de Asturias (Oviedo)

Víctor Asensi, Eulalia Valle, María Eugenia Rivas Carmenado, Tomas Suarez-Zarracina Secades, Laura Pérez Is.

Hospital Universitario 12 de Octubre (Madrid)

Rafael Rubio, Federico Pulido, Otilia Bisbal, Asunción Hernando, Lourdes Domínguez, David Rial Crestelo, Laura Bermejo, Mireia Santacreu.

Hospital Universitario de Donostia (Donostia-San Sebastián)

José Antonio Iribarren, Julio Arrizabalaga, María José Aramburu, Xabier Camino, Francisco Rodríguez-Arrondo, Miguel Ángel von Wichmann, Lidia Pascual Tomé, Miguel Ángel Goenaga, Mª Jesús Bustinduy, Harkaitz Azkune, Maialen Ibarguren, Aitziber Lizardi, Xabier Kortajarena, Mª Pilar Carmona Oyaga, Maitane Umerez Igartua.

Hospital General Universitario De Elche (Elche)

Félix Gutiérrez, Mar Masiá, Sergio Padilla, Catalina Robledano, Joan Gregori Colomé, Araceli Adsuar, Rafael Pascual, Marta Fernández, José Alberto García, Xavier Barber, Vanessa Agullo Re, Javier Garcia Abellan, Reyes Pascual Pérez, María Roca.

Hospital General Universitario Gregorio Marañón (Madrid)

Juan Berenguer, Juan Carlos López Bernaldo de Quirós, Isabel Gutiérrez, Margarita Ramírez, Belén Padilla, Paloma Gijón, Teresa Aldamiz-Echevarría, Francisco Tejerina, Francisco José Parras, Pascual Balsalobre, Cristina Diez, Leire Pérez Latorre., Chiara Fanciulli.

Hospital Universitari de Tarragona Joan XXIII (Tarragona)

Francesc Vidal, Joaquín Peraire, Consuelo Viladés, Sergio Veloso, Montserrat Vargas, Montserrat Olona, Anna Rull, Esther Rodríguez-Gallego, Verónica Alba., Alfonso Javier Castellanos, Miguel López-Dupla.

Hospital Universitario y Politécnico de La Fe (Valencia)

Marta Montero Alonso, José López Aldeguer, Marino Blanes Juliá, María Tasias Pitarch, Iván Castro Hernández, Eva Calabuig Muñoz, Sandra Cuéllar Tovar, Miguel Salavert Lletí, Juan Fernández Navarro.

Hospital Universitario La Paz/IdiPAZ

Juan González-Garcia, Francisco Arnalich, José Ramón Arribas, Jose Ignacio Bernardino de la Serna, Juan Miguel Castro, Ana Delgado Hierro, Luis Escosa, Pedro Herranz, Víctor Hontañón, Silvia García-Bujalance, Milagros García López-Hortelano, Alicia González-Baeza, Maria Luz Martín-Carbonero, Mario Mayoral, Maria Jose Mellado, Rafael Esteban Micán, Rocio Montejano, María Luisa Montes, Victoria Moreno, Ignacio Pérez-Valero, Guadalupe Rúa Cebrián, Berta Rodés, Talia Sainz, Elena Sendagorta, Natalia Stella Alcáriz, Eulalia Valencia.

Hospital Universitari MutuaTerrassa (Terrasa)

David Dalmau, Angels Jaén, Montse Sanmartí, Mireia Cairó, Javier Martinez-Lacasa, Pablo Velli, Roser Font, Marina Martinez, Francesco Aiello.

Hospital Universitario de La Princesa (Madrid)

Ignacio de los Santos, Jesus Sanz Sanz, Ana Salas Aparicio, Cristina Sarria Cepeda, Lucio Garcia-Fraile Fraile, Enrique Martín Gayo.

Hospital Universitario Ramón y Cajal (Madrid)

Santiago Moreno, José Luis Casado Osorio, Fernando Dronda Nuñez, Ana Moreno Zamora, Maria Jesús Pérez Elías, Carolina Gutiérrez, Nadia Madrid, Santos del Campo Terrón, Sergio Serrano Villar, Maria Jesús Vivancos Gallego, Javier Martínez Sanz, Usua Anxa Urroz, Tamara Velasco, Alejandro Vallejo.

Hospital General Universitario Reina Sofía (Murcia)

Enrique Bernal, Alfredo Cano Sanchez, Antonia Alcaraz García, Joaquín Bravo Urbieta, Angeles Muñoz Perez, Maria Jose Alcaraz, Maria del Carmen Villalba.

Hospital Nuevo San Cecilio (Granada)

Federico García, José Hernández Quero, Leopoldo Muñoz Medina, Marta Alvarez, Natalia Chueca, David Vinuesa García, Clara Martinez-Montes., Carlos Guerrero Beltran, Adolfo de Salazar Gonzalerz, Ana Fuentes Lopez.

Centro Sanitario Sandoval (Madrid)

Montserrat Raposo Utrilla, Jorge Del Romero, Carmen Rodríguez, Teresa Puerta, Juan Carlos Carrió, Mar Vera, Juan Ballesteros, Oskar Ayerdi.

Hospital Universitario Son Espases (Palma de Mallorca)

Melchor Riera, María Peñaranda, Mª Angels Ribas, Antoni A Campins, Carmen Vidal, Francisco Fanjul, Javier Murillas, Francisco Homar, Helem H Vilchez, Maria Luisa Martin, Antoni Payeras.

Hospital Universitario Virgen de la Victoria (Málaga)

Jesús Santos, Crisitina Gómez Ayerbe, Isabel Viciana, Rosario Palacios, Carmen Pérez López, Carmen Maria Gonzalez-Domenec.

Hospital Universitario Virgen del Rocío (Sevilla)

Pompeyo Viciana, Nuria Espinosa, Luis Fernando López-Cortés.

Hospital Universitario de Bellvitge (Hospitalet de Llobregat)

Daniel Podzamczer, Arkaitz Imaz, Juan Tiraboschi, Ana Silva, María Saumoy, Paula Prieto.

Hospital Costa del Sol (Marbella)

Julián Olalla Sierra, Javier Pérez Stachowski., Alfonso del Arco, Javier de la torre, José Luis Prada, José María García de Lomas Guerrero.

Hospital General Universitario Santa Lucía (Cartagena)

Onofre Juan Martínez, Francisco Jesús Vera, Lorena Martínez, Josefina García, Begoña Alcaraz, Amaya Jimeno.

Complejo Hospitalario Universitario a Coruña (Chuac) (A Coruña)

Angeles Castro Iglesias, Berta Pernas Souto, Alvaro Mena de Cea.

Hospital Universitario Virgen de la Arrixaca (El Palmar)

Carlos Galera, Helena Albendin, Aurora Pérez, Asunción Iborra, Antonio Moreno, Maria Angustias Merlos, Asunción Vidal, Marisa Meca.

Hospital Universitario Infanta Sofia (San Sebastian de los Reyes)

Inés Suárez-García, Eduardo Malmierca, Patricia González-Ruano, Dolores Martín Rodrigo, Mª Pilar Ruiz Seco.

Hospital Universitario Príncipe de Asturias (Alcalá de Henares)

José Sanz Moreno, Alberto Arranz Caso, Cristina Hernández Gutiérrez, María Novella Mena.

Hospital Clínico Universitario de Valencia (València)

María Jos Galindo Puerto, Ramón Fernando Vilalta, Ana Ferrer Ribera.

Hospital Reina Sofía (Córdoba)

Antonio Rivero Román, Antonio Rivero Juárez, Pedro López López, Isabel Machuca Sánchez, Mario Frias Casas, Angela Camacho Espejo.

Hospital Universitario Severo Ochoa (Leganés)

Miguel Cervero Jiménez, Rafael Torres Perea.

Nuestra Señora de Valme (Sevilla)

Juan A Pineda, Pilar Rincón Mayo, Juan Macias Sanchez, Nicolas Merchante Gutierrez, Luis Miguel Real, Anais Corma Gomez, Marta Fernandez Fuertes, Alejandro Gonzalez-Serna.
